# Supplementary material for: Is there an ex-ante moral hazard on Indonesia’s health insurance? An impact analysis on household waste management behavior
Source: PLoS One. 2022 Dec 15;17(12):e0276521. doi: 10.1371/journal.pone.0276521 (PMC9754289; doi:10.1371/journal.pone.0276521)
Supplement: S1 Appendix — (DOCX) [file pone.0276521.s001.docx]

**Appendices**

**S7 Table. Chronic Disease and Income Distribution**

**Panel A. IFLS 2007: Tabulation of Chronic Diseases and Income (per quantile)**

| **Chronic Disease (2007)** | **Total Household Income (2007) by quantiles** | | | | | | | | | |
| --- | --- | --- | --- | --- | --- | --- | --- | --- | --- | --- |
|  | **<Rp3.400.000** | | **Rp3.400.001 - Rp6.500.000** | | **Rp6.500.001 - Rp11.400.000** | | **Rp11.400.001 - Rp20.000.000** | | **>Rp20.000.000** | |
|  | **%** | **n** | **%** | **n** | **%** | **n** | **%** | **n** | **%** | **n** |
| Hypertension | 14.01% | 162 | 13.93% | 133 | 13.51% | 117 | 16.19% | 148 | 19.10% | 208 |
| Diabetes | 1.30% | 15 | 1.47% | 14 | 2.08% | 18 | 2.41% | 22 | 5.97% | 65 |
| Tuberculosis | 0.35% | 4 | 0.52% | 5 | 0.23% | 2 | 1.42% | 13 | 0.46% | 5 |
| Asthma | 3.72% | 43 | 2.93% | 28 | 3.58% | 31 | 3.17% | 29 | 2.48% | 27 |
| Other lung conditions | 2.34% | 27 | 1.47% | 14 | 2.31% | 20 | 2.52% | 23 | 2.75% | 30 |
| Heart attack / coronary heart | 1.56% | 18 | 1.88% | 18 | 1.27% | 11 | 2.63% | 24 | 2.57% | 28 |
| Liver | 0.26% | 3 | 0.31% | 3 | 0.35% | 3 | 1.09% | 10 | 0.92% | 10 |
| Stroke | 0.43% | 5 | 0.84% | 8 | 1.04% | 9 | 0.44% | 4 | 1.10% | 12 |
| Cancer / malignant tumor | 0.17% | 2 | 0.52% | 5 | 0.35% | 3 | 0.22% | 2 | 0.28% | 3 |
| Arthritis / rheumatism | 10.73% | 124 | 10.37% | 99 | 9.01% | 78 | 8.10% | 74 | 7.62% | 83 |
| Uric Acid/Gout | 5.02% | 58 | 4.08% | 39 | 4.39% | 38 | 6.46% | 59 | 9.83% | 107 |
| **Total Respondents (by income)** |  | 1,156.00 |  | 955 |  | 866 |  | 914 |  | 1,089.00 |
|  |  |  |  |  |  |  |  |  |  |  |

**Panel B. IFLS 2014: Tabulation of Chronic Diseases and Income (per quantile)**

| **Chronic Disease (2014)** | **Total Household Income (2014) by quantiles** | | | | | | | | | |
| --- | --- | --- | --- | --- | --- | --- | --- | --- | --- | --- |
|  | **<Rp7.000.000** | | **Rp7.000.001 - Rp15.000.000** | | **Rp15.000.001 - Rp25.350.000** | | **Rp25.250.001 - Rp46.800.000** | | **>Rp46.800.000** | |
|  | **%** | **n** | **%** | **n** | **%** | **n** | **%** | **n** | **%** | **n** |
| Hypertension | 15.33% | 259 | 12.81% | 223 | 13.09% | 211 | 13.67% | 228 | 16.25% | 268 |
| Diabetes | 2.78% | 47 | 3.10% | 54 | 2.73% | 44 | 3.42% | 57 | 4.85% | 80 |
| Tuberculosis | 1.24% | 21 | 0.80% | 14 | 1.18% | 19 | 1.50% | 25 | 1.27% | 21 |
| Asthma | 2.78% | 47 | 1.84% | 32 | 2.42% | 39 | 2.34% | 39 | 2.97% | 49 |
| Other lung conditions | 1.60% | 27 | 2.13% | 37 | 1.67% | 27 | 1.50% | 25 | 1.64% | 27 |
| Heart attack / coronary heart | 2.01% | 34 | 1.55% | 27 | 2.30% | 37 | 1.80% | 30 | 2.49% | 41 |
| Liver | 1.01% | 17 | 0.86% | 15 | 1.49% | 24 | 1.02% | 17 | 1.58% | 26 |
| Stroke | 0.71% | 12 | 1.21% | 21 | 0.68% | 11 | 0.54% | 9 | 1.15% | 19 |
| Cancer / malignant tumor | 0.83% | 14 | 0.29% | 5 | 0.62% | 10 | 0.48% | 8 | 0.42% | 7 |
| Arthritis / rheumatism | 8.11% | 137 | 7.47% | 130 | 5.71% | 92 | 5.10% | 85 | 7.46% | 123 |
| High Cholesterol | 3.02% | 51 | 3.16% | 55 | 4.65% | 75 | 5.52% | 92 | 11.04% | 182 |
| Prostate Illness | 0.65% | 11 | 0.69% | 12 | 0.99% | 16 | 0.78% | 13 | 0.49% | 8 |
| Kidney disease | 1.60% | 27 | 1.72% | 30 | 1.49% | 24 | 1.86% | 31 | 2.43% | 40 |
| Digestive disease | 10.12% | 171 | 9.48% | 165 | 9.68% | 156 | 12.77% | 213 | 13.34% | 220 |
| Psychiatric Problem | 0.06% | 1 | 0.06% | 1 | 0.12% | 2 | 0.06% | 1 | 0.55% | 9 |
| Memory-related disease | 0.24% | 4 | 0.34% | 6 | 0.06% | 1 | 0.18% | 3 | 0.18% | 3 |
| **Total Respondents (by income)** |  | 1,689.00 |  | 1,741.00 |  | 1,612.00 |  | 1,668.00 |  | 1,649.00 |
|  |  |  |  |  |  |  |  |  |  |  |

**S8 Table. Acute Disease and Income Distribution**

**Panel A. IFLS 2007: Acute illness during the last 4 weeks**

| **Acute Disease (2007)** | **Total Household Income (2007) by quantiles** | | | | | | | | | |
| --- | --- | --- | --- | --- | --- | --- | --- | --- | --- | --- |
|  | **<Rp3.400.000** | | **Rp3.400.001 - Rp6.500.000** | | **Rp6.500.001 - Rp11.400.000** | | **Rp11.400.001 - Rp20.000.000** | | **>Rp20.000.000** | |
|  | **%** | **n** | **%** | **n** | **%** | **n** | **%** | **n** | **%** | **n** |
| Headache | 52.11% | 989 | 50.82% | 961 | 50.60% | 965 | 52.45% | 996 | 48.83% | 916 |
| Runny nose | 36.62% | 695 | 37.55% | 710 | 38.75% | 739 | 37.23% | 707 | 35.07% | 658 |
| Cough | 32.35% | 614 | 29.98% | 567 | 30.83% | 588 | 29.38% | 558 | 28.41% | 533 |
| Dry cough | 19.81% | 376 | 18.24% | 345 | 17.88% | 341 | 14.96% | 284 | 16.10% | 302 |
| Cough with phlegm | 13.33% | 253 | 12.64% | 239 | 13.37% | 255 | 14.90% | 283 | 13.01% | 244 |
| Bloody cough | 0.58% | 11 | 0.53% | 10 | 0.21% | 4 | 0.47% | 9 | 0.27% | 5 |
| **Difficulty breathing** | 8.27% | 157 | 6.87% | 130 | 6.87% | 131 | 5.95% | 113 | 5.12% | 96 |
| Wheezing | 2.74% | 52 | 1.80% | 34 | 2.31% | 44 | 1.37% | 26 | 1.71% | 32 |
| Short, rapid breath | 5.85% | 111 | 5.18% | 98 | 4.98% | 95 | 4.58% | 87 | 3.68% | 69 |
| Fever | 17.28% | 328 | 17.87% | 338 | 17.62% | 336 | 16.22% | 308 | 13.11% | 246 |
| **Stomachache** | 15.54% | 295 | 16.45% | 311 | 17.25% | 329 | 18.59% | 353 | 16.90% | 317 |
| **Nausea/vomiting** | 6.43% | 122 | 7.35% | 139 | 9.44% | 180 | 10.06% | 191 | 8.90% | 167 |
| **Diarrhea** | 5.80% | 110 | 6.03% | 114 | 6.61% | 126 | 5.90% | 112 | 6.88% | 129 |
| **Diarrhea mixed with blood** | 0.47% | 9 | 0.26% | 5 | 0.42% | 8 | 0.42% | 8 | 0.16% | 3 |
| **Diarrhea mixed with mucous** | 2.11% | 40 | 2.38% | 45 | 2.46% | 47 | 2.37% | 45 | 3.52% | 66 |
| **Diarrhea with pale liquid** | 3.21% | 61 | 3.49% | 66 | 3.67% | 70 | 3.05% | 58 | 3.41% | 64 |
| **Skin infection (boil, abscess itching)** | 9.11% | 173 | 8.73% | 165 | 8.81% | 168 | 8.06% | 153 | 7.62% | 143 |
| Eye infection | 5.95% | 113 | 3.91% | 74 | 5.40% | 103 | 4.48% | 85 | 4.58% | 86 |
| Toothache | 12.64% | 240 | 11.95% | 226 | 13.11% | 250 | 11.48% | 218 | 10.45% | 196 |
| Swollen legs | 2.32% | 44 | 2.27% | 43 | 1.68% | 32 | 1.95% | 37 | 1.87% | 35 |
| Cold sores | 7.64% | 145 | 8.25% | 156 | 10.17% | 194 | 11.59% | 220 | 11.30% | 212 |
| **Total Respondents (by income)** |  | 1,898.00 |  | 1,891 |  | 1,907 |  | 1,899 |  | 1,876.00 |

**Panel B. IFLS 2007: Acute illness during the last 4 weeks**

| **Acute Disease (2014)** | **Total Household Income (2014) by quantiles** | | | | | | | | | |
| --- | --- | --- | --- | --- | --- | --- | --- | --- | --- | --- |
|  | **<Rp7.000.000** | | **Rp7.000.001 - Rp15.000.000** | | **Rp15.000.001 - Rp25.350.000** | | **Rp25.250.001 - Rp46.800.000** | | **>Rp46.800.000** | |
|  | **%** | **n** | **%** | **n** | **%** | **n** | **%** | **n** | **%** | **n** |
| Headache | 54.16% | 925 | 56.53% | 995 | 58.09% | 977 | 58.28% | 992 | 54.45% | 923 |
| Runny nose | 44.50% | 760 | 46.31% | 815 | 44.53% | 749 | 44.89% | 764 | 44.60% | 756 |
| Cough | 41.39% | 707 | 41.99% | 739 | 40.84% | 687 | 39.31% | 669 | 39.17% | 664 |
| Dry cough | 22.13% | 378 | 21.19% | 373 | 20.99% | 353 | 19.74% | 336 | 19.82% | 336 |
| Cough with phlegm | 21.90% | 374 | 22.84% | 402 | 22.29% | 375 | 21.97% | 374 | 21.77% | 369 |
| Bloody cough | 0.41% | 7 | 0.23% | 4 | 0.36% | 6 | 0.53% | 9 | 0.12% | 2 |
| **Difficulty breathing** | 9.43% | 161 | 7.73% | 136 | 7.91% | 133 | 7.52% | 128 | 7.61% | 129 |
| Wheezing | 3.75% | 64 | 2.95% | 52 | 2.26% | 38 | 2.47% | 42 | 2.60% | 44 |
| Short, rapid breath | 7.08% | 121 | 6.02% | 106 | 6.12% | 103 | 6.17% | 105 | 5.60% | 95 |
| Fever | 19.56% | 334 | 20.40% | 359 | 20.51% | 345 | 20.86% | 355 | 20.59% | 349 |
| **Stomachache** | 23.59% | 403 | 24.20% | 426 | 25.03% | 421 | 27.56% | 469 | 26.19% | 444 |
| **Nausea/vomiting** | 12.30% | 210 | 12.05% | 212 | 12.60% | 212 | 13.98% | 238 | 13.81% | 234 |
| **Diarrhea** | 9.48% | 162 | 9.89% | 174 | 10.34% | 174 | 10.46% | 178 | 9.03% | 153 |
| **Diarrhea mixed with blood** | 0.76% | 13 | 0.11% | 2 | 0.59% | 10 | 0.65% | 11 | 0.65% | 11 |
| **Diarrhea mixed with mucous** | 3.81% | 65 | 3.69% | 65 | 3.09% | 52 | 3.88% | 66 | 2.77% | 47 |
| **Diarrhea with pale liquid** | 4.92% | 84 | 5.80% | 102 | 6.00% | 101 | 4.05% | 69 | 4.48% | 76 |
| **Skin infection (boil, abcess itching)** | 16.80% | 287 | 15.40% | 271 | 14.74% | 248 | 13.87% | 236 | 13.27% | 225 |
| Eye infection | 8.72% | 149 | 6.70% | 118 | 5.59% | 94 | 5.88% | 100 | 5.07% | 86 |
| Toothache | 17.27% | 295 | 16.14% | 284 | 14.45% | 243 | 16.22% | 276 | 14.10% | 239 |
| Swollen legs | 4.51% | 77 | 4.49% | 79 | 3.51% | 59 | 4.17% | 71 | 3.54% | 60 |
| Cold sores | 13.29% | 227 | 15.57% | 274 | 15.93% | 268 | 17.98% | 306 | 16.64% | 282 |
| **Total Respondents (by income)** |  | 1,708.00 |  | 1,760.00 |  | 1,682.00 |  | 1,702.00 |  | 1,695.00 |

**S9 Table. Descriptive Statistics, IFLS 2007 and 2014**

|  | (1) | (2) | (3) | (4) | (5) | (6) | (7) | (8) | (9) | (10) |
| --- | --- | --- | --- | --- | --- | --- | --- | --- | --- | --- |
| VARIABLES |  | | | | | |  | |  | |
|  | Overall Sample | | Urban | | Rural | | 2007 | | 2014 | |
|  | N | mean | N | mean | N | mean | N | mean | N | mean |
| **Household Characteristics** |  |  |  |  |  |  |  |  |  |  |
| Subsidized Insurance (PBI) = 1 | 9,369 | 0.191 | 4,681 | 0.185 | 4,688 | 0.197 | 4,870 | 0.190 | 4,499 | 0.191 |
| Years of Education | 9,369 | 8.520 | 4,681 | 10.17 | 4,688 | 6.877 | 4,870 | 8.250 | 4,499 | 8.814 |
| Risk Preferences = 1 | 9,369 | 0.260 | 4,681 | 0.267 | 4,688 | 0.254 | 4,870 | 0.277 | 4,499 | 0.242 |
| Risk Preferences = 2 | 9,369 | 0.077 | 4,681 | 0.082 | 4,688 | 0.074 | 4,870 | 0.055 | 4,499 | 0.102 |
| Risk Preferences = 3 | 9,369 | 0.099 | 4,681 | 0.106 | 4,688 | 0.092 | 4,870 | 0.082 | 4,499 | 0.118 |
| Risk Preferences = 4 | 9,369 | 0.208 | 4,681 | 0.209 | 4,688 | 0.206 | 4,870 | 0.185 | 4,499 | 0.232 |
|  |  |  |  |  |  |  |  |  |  |  |
| **Environment and Community** |  |  |  |  |  |  |  |  |  |  |
| System of drain/sewage channel = 1 | 9,369 | 0.519 | 4,681 | 0.626 | 4,688 | 0.403 | 4,870 | 0.525 | 4,499 | 0.513 |
| Proportion of Garbage Collector in District Level | 9,369 | 0.272 | 4,681 | 0.423 | 4,688 | 0.122 | 4,870 | 0.241 | 4,499 | 0.307 |
| Cleaning Routine in Community = 1 | 9,369 | 0.496 | 4,681 | 0.490 | 4,688 | 0.501 | 4,870 | 0.516 | 4,499 | 0.473 |
|  |  |  |  |  |  |  |  |  |  |  |
| **Location and Density** |  |  |  |  |  |  |  |  |  |  |
| Urban = 1 | 9,369 | 0.499 | 4,681 | 1 | 4,688 | 0 | 4,870 | 0.468 | 4,499 | 0.534 |
| Jawa Island | 9,369 | 0.560 | 4,681 | 0.614 | 4,688 | 0.508 | 4,870 | 0.569 | 4,499 | 0.553 |
| Bali, NTT, NTB Island | 9,369 | 0.116 | 4,681 | 0.122 | 4,688 | 0.110 | 4,870 | 0.113 | 4,499 | 0.119 |
| Kalimantan Island | 9,369 | 0.066 | 4,681 | 0.054 | 4,688 | 0.077 | 4,870 | 0.064 | 4,499 | 0.067 |
| Sulawesi Island | 9,369 | 0.046 | 4,681 | 0.043 | 4,688 | 0.048 | 4,870 | 0.045 | 4,499 | 0.046 |
| Log of Population Density (People/Km2) | 9,369 | 6.593 | 4,681 | 7.259 | 4,688 | 5.927 | 4,870 | 6.583 | 4,499 | 6.603 |

**S10 Table. Main Regressions (Full Model)**

|  | (1) | (2) | (3) | (4) | (5) | (6) | (7) | (8) | (9) | (10) | (11) | (12) | (13) | (14) | (15) |
| --- | --- | --- | --- | --- | --- | --- | --- | --- | --- | --- | --- | --- | --- | --- | --- |
|  | Disposed in Trash Can | Disposed in Trash Can | Disposed in Trash Can | Disposed in Trash Can | Disposed in Trash Can | Burned | Burned | Burned | Burned | Burned | Disposed in Land, Water, Yard | Disposed in Land, Water, Yard | Disposed in Land, Water, Yard | Disposed in Land, Water, Yard | Disposed in Land, Water, Yard |
| Subsidized Insurance = 1 | -0.102^***^ | -0.003 | -0.004 | -0.006 | 0.011 | -0.044^**^ | -0.082^***^ | -0.073^***^ | -0.059^***^ | -0.053^***^ | 0.115^***^ | 0.063^***^ | 0.075^***^ | 0.068^***^ | 0.055^***^ |
|  | (0.017) | (0.016) | (0.013) | (0.013) | (0.013) | (0.017) | (0.018) | (0.017) | (0.017) | (0.017) | (0.014) | (0.014) | (0.014) | (0.014) | (0.015) |
|  |  |  |  |  |  |  |  |  |  |  |  |  |  |  |  |
| POST = 1 | 0.054^***^ | 0.044^***^ | 0.007 | 0.003 | -0.003 | 0.035^***^ | 0.042^***^ | 0.079^***^ | 0.072^***^ | 0.066^***^ | -0.102^***^ | -0.096^***^ | -0.081^***^ | -0.078^***^ | -0.061^***^ |
|  | (0.010) | (0.009) | (0.007) | (0.014) | (0.015) | (0.011) | (0.011) | (0.010) | (0.020) | (0.021) | (0.009) | (0.009) | (0.009) | (0.019) | (0.020) |
|  |  |  |  |  |  |  |  |  |  |  |  |  |  |  |  |
| Subsidized Insurance X POST | -0.023 | -0.025 | -0.038^**^ | -0.037^**^ | -0.039^**^ | 0.056^**^ | 0.053^**^ | 0.058^**^ | 0.056^**^ | 0.038 | -0.007 | -0.004 | 0.003 | -0.002 | 0.006 |
|  | (0.024) | (0.023) | (0.018) | (0.018) | (0.018) | (0.025) | (0.025) | (0.024) | (0.024) | (0.024) | (0.021) | (0.020) | (0.021) | (0.021) | (0.021) |
|  |  |  |  |  |  |  |  |  |  |  |  |  |  |  |  |
| Years of Education |  | 0.025^***^ | 0.010^***^ | 0.008^***^ | 0.007^***^ |  | -0.012^***^ | -0.002^*^ | -0.000 | -0.002 |  | -0.016^***^ | -0.010^***^ | -0.010^***^ | -0.008^***^ |
|  |  | (0.001) | (0.001) | (0.001) | (0.001) |  | (0.001) | (0.001) | (0.001) | (0.001) |  | (0.001) | (0.001) | (0.001) | (0.001) |
|  |  |  |  |  |  |  |  |  |  |  |  |  |  |  |  |
| Degree of Risk Preference = 1 |  | 0.015 | -0.001 | -0.001 | -0.003 |  | 0.001 | 0.013 | 0.013 | 0.010 |  | -0.017^*^ | -0.009 | -0.009 | -0.006 |
|  |  | (0.011) | (0.009) | (0.008) | (0.008) |  | (0.012) | (0.012) | (0.012) | (0.012) |  | (0.010) | (0.011) | (0.011) | (0.011) |
|  |  |  |  |  |  |  |  |  |  |  |  |  |  |  |  |
| Degree of Risk Preference = 2 |  | -0.011 | -0.016 | -0.010 | -0.017 |  | -0.001 | 0.009 | 0.001 | 0.004 |  | 0.003 | 0.007 | 0.011 | 0.015 |
|  |  | (0.016) | (0.013) | (0.013) | (0.013) |  | (0.019) | (0.019) | (0.019) | (0.019) |  | (0.017) | (0.017) | (0.017) | (0.017) |
|  |  |  |  |  |  |  |  |  |  |  |  |  |  |  |  |
| Degree of Risk Preference = 3 |  | 0.001 | -0.014 | -0.015 | -0.020^*^ |  | 0.002 | 0.013 | 0.015 | 0.019 |  | -0.001 | 0.002 | 0.001 | 0.001 |
|  |  | (0.015) | (0.012) | (0.011) | (0.012) |  | (0.017) | (0.017) | (0.017) | (0.017) |  | (0.015) | (0.015) | (0.015) | (0.016) |
|  |  |  |  |  |  |  |  |  |  |  |  |  |  |  |  |
| Degree of Risk Preference = 4 |  | -0.014 | -0.005 | -0.003 | -0.007 |  | -0.007 | -0.011 | -0.007 | -0.005 |  | 0.022^*^ | 0.018 | 0.012 | 0.013 |
|  |  | (0.011) | (0.009) | (0.009) | (0.009) |  | (0.013) | (0.013) | (0.013) | (0.013) |  | (0.012) | (0.012) | (0.012) | (0.012) |
|  |  |  |  |  |  |  |  |  |  |  |  |  |  |  |  |
| Cleaning Routine in Community = 1 |  |  | -0.009 | -0.004 | -0.000 |  |  | 0.003 | -0.001 | -0.006 |  |  | 0.002 | -0.000 | 0.001 |
|  |  |  | (0.007) | (0.007) | (0.007) |  |  | (0.009) | (0.009) | (0.010) |  |  | (0.008) | (0.008) | (0.009) |
|  |  |  |  |  |  |  |  |  |  |  |  |  |  |  |  |
| System of drain/sewage channel = 1 |  |  | 0.116^***^ | 0.101^***^ | 0.097^***^ |  |  | -0.056^***^ | -0.057^***^ | -0.064^***^ |  |  | -0.051^***^ | -0.035^***^ | -0.028^***^ |
|  |  |  | (0.007) | (0.007) | (0.007) |  |  | (0.010) | (0.010) | (0.010) |  |  | (0.009) | (0.009) | (0.009) |
|  |  |  |  |  |  |  |  |  |  |  |  |  |  |  |  |
| Proportion of Garbage Collector in District Level |  |  | 0.579^***^ | 0.489^***^ | 0.470^***^ |  |  | -0.680^***^ | -0.620^***^ | -0.601^***^ |  |  | -0.310^***^ | -0.274^***^ | -0.269^***^ |
|  |  |  | (0.013) | (0.015) | (0.015) |  |  | (0.022) | (0.026) | (0.026) |  |  | (0.022) | (0.026) | (0.027) |
|  |  |  |  |  |  |  |  |  |  |  |  |  |  |  |  |
| Log of Population Density (People/Km2) |  |  | 0.001 | -0.016^***^ | -0.016^***^ |  |  | 0.021^***^ | 0.037^***^ | 0.035^***^ |  |  | -0.017^***^ | -0.017^***^ | -0.015^***^ |
|  |  |  | (0.003) | (0.003) | (0.003) |  |  | (0.004) | (0.006) | (0.006) |  |  | (0.004) | (0.005) | (0.005) |
|  |  |  |  |  |  |  |  |  |  |  |  |  |  |  |  |
| Urban = 1 |  |  |  | 0.158^***^ | 0.147^***^ |  |  |  | -0.102^***^ | -0.101^***^ |  |  |  | -0.050^***^ | -0.045^***^ |
|  |  |  |  | (0.007) | (0.008) |  |  |  | (0.011) | (0.011) |  |  |  | (0.010) | (0.010) |
|  |  |  |  |  |  |  |  |  |  |  |  |  |  |  |  |
| Island = Java Island |  |  |  | 0.029^***^ | 0.035^***^ |  |  |  | -0.099^***^ | -0.101^***^ |  |  |  | 0.073^***^ | 0.070^***^ |
|  |  |  |  | (0.009) | (0.009) |  |  |  | (0.014) | (0.014) |  |  |  | (0.012) | (0.012) |
|  |  |  |  |  |  |  |  |  |  |  |  |  |  |  |  |
| Island = Bali, NTT, NTB, Island |  |  |  | 0.025^**^ | 0.029^**^ |  |  |  | -0.182^***^ | -0.186^***^ |  |  |  | 0.184^***^ | 0.185^***^ |
|  |  |  |  | (0.012) | (0.012) |  |  |  | (0.017) | (0.017) |  |  |  | (0.015) | (0.016) |
|  |  |  |  |  |  |  |  |  |  |  |  |  |  |  |  |
| Island = Kalimantan Island |  |  |  | 0.019 | 0.017 |  |  |  | -0.101^***^ | -0.117^***^ |  |  |  | 0.108^***^ | 0.124^***^ |
|  |  |  |  | (0.014) | (0.014) |  |  |  | (0.021) | (0.022) |  |  |  | (0.019) | (0.019) |
|  |  |  |  |  |  |  |  |  |  |  |  |  |  |  |  |
| Island = Sulawesi Island |  |  |  | 0.023 | 0.027^*^ |  |  |  | -0.135^***^ | -0.163^***^ |  |  |  | 0.121^***^ | 0.143^***^ |
|  |  |  |  | (0.017) | (0.016) |  |  |  | (0.023) | (0.024) |  |  |  | (0.021) | (0.022) |
|  |  |  |  |  |  |  |  |  |  |  |  |  |  |  |  |
| HH Head Age |  |  |  |  | -0.000 |  |  |  |  | -0.000 |  |  |  |  | 0.000 |
|  |  |  |  |  | (0.000) |  |  |  |  | (0.000) |  |  |  |  | (0.000) |
|  |  |  |  |  |  |  |  |  |  |  |  |  |  |  |  |
| Per Capita Expenditure |  |  |  |  | 0.002^***^ |  |  |  |  | -0.002^***^ |  |  |  |  | -0.001 |
|  |  |  |  |  | (0.000) |  |  |  |  | (0.001) |  |  |  |  | (0.001) |
|  |  |  |  |  |  |  |  |  |  |  |  |  |  |  |  |
| Household Size |  |  |  |  | 0.001 |  |  |  |  | 0.003 |  |  |  |  | -0.004 |
|  |  |  |  |  | (0.002) |  |  |  |  | (0.003) |  |  |  |  | (0.003) |
|  |  |  |  |  |  |  |  |  |  |  |  |  |  |  |  |
| HH Head is Male |  |  |  |  | 0.002 |  |  |  |  | -0.017 |  |  |  |  | 0.010 |
|  |  |  |  |  | (0.011) |  |  |  |  | (0.016) |  |  |  |  | (0.015) |
|  |  |  |  |  |  |  |  |  |  |  |  |  |  |  |  |
| HH Head is Married |  |  |  |  | -0.026^**^ |  |  |  |  | 0.012 |  |  |  |  | 0.029^*^ |
|  |  |  |  |  | (0.012) |  |  |  |  | (0.017) |  |  |  |  | (0.016) |
|  |  |  |  |  |  |  |  |  |  |  |  |  |  |  |  |
| Wall is Bamboo |  |  |  |  | -0.056^**^ |  |  |  |  | 0.047^**^ |  |  |  |  | -0.008 |
|  |  |  |  |  | (0.022) |  |  |  |  | (0.023) |  |  |  |  | (0.019) |
|  |  |  |  |  |  |  |  |  |  |  |  |  |  |  |  |
| Floor is Not Soil |  |  |  |  | 0.067^***^ |  |  |  |  | 0.051^**^ |  |  |  |  | -0.031^*^ |
|  |  |  |  |  | (0.024) |  |  |  |  | (0.021) |  |  |  |  | (0.017) |
|  |  |  |  |  |  |  |  |  |  |  |  |  |  |  |  |
| HH has Vehicle |  |  |  |  | -0.003 |  |  |  |  | 0.046^***^ |  |  |  |  | -0.042^***^ |
|  |  |  |  |  | (0.008) |  |  |  |  | (0.011) |  |  |  |  | (0.010) |
|  |  |  |  |  |  |  |  |  |  |  |  |  |  |  |  |
| HH has Electronic Asset |  |  |  |  | -0.038^**^ |  |  |  |  | 0.050^***^ |  |  |  |  | -0.008 |
|  |  |  |  |  | (0.016) |  |  |  |  | (0.019) |  |  |  |  | (0.016) |
|  |  |  |  |  |  |  |  |  |  |  |  |  |  |  |  |
| HH has electricity |  |  |  |  | 0.019 |  |  |  |  | 0.041 |  |  |  |  | -0.002 |
|  |  |  |  |  | (0.034) |  |  |  |  | (0.031) |  |  |  |  | (0.026) |
|  |  |  |  |  |  |  |  |  |  |  |  |  |  |  |  |
| Has no toilet |  |  |  |  | -0.074^***^ |  |  |  |  | -0.043^***^ |  |  |  |  | 0.061^***^ |
|  |  |  |  |  | (0.017) |  |  |  |  | (0.016) |  |  |  |  | (0.014) |
| *N* | 10,735 | 10,651 | 9,997 | 9,992 | 9,626 | 10,735 | 10,651 | 9,997 | 9,992 | 9,626 | 10,735 | 10,651 | 9,997 | 9,992 | 9,626 |
