# Supplementary material for: Is there an ex-ante moral hazard on Indonesia’s health insurance? An impact analysis on household waste management behavior
Source: PLoS One. 2022 Dec 15;17(12):e0276521. doi: 10.1371/journal.pone.0276521 (PMC9754289; doi:10.1371/journal.pone.0276521)
Supplement: Attachment — Submitted filename: Response to Reviewers_Revision 2.docx [file pone.0276521.s002.docx]

**Response to Referees**

Dear Editor and Referees

We thank the referees for providing additional comments for the revision. We have tried to respond to all the comments and accommodate the suggestions made by the reviewer into our substantially further revised manuscript.

The significant changes that we made from the referee’s comments is that we strengthen the argumentation to back up the claim that the impact of our variable of interest is not affected by group specific characteristics. As a respond to the suggestion, we double check the estimates and do the necessary correction and revision toward the updated findings. We also add several further details and refinement on various part of paragraph and texts reflecting the revised finding and conclusion. A point-by-point response to the reviewer follows below.

| **Reviewer’s Comments** | **Authors’ responses** |
| --- | --- |
| The study has been well written, so does the introduction section. It provides clear argument about the importance of the study. However, I would like to recommend one small thing for this section. The authors have mention that they have found an evidence of ex-ante moral hazard in the subsidized health insurance recipients on how they manage their household waste. The insured households are more likely to burn their domestic waste than the uninsured. How do we know that this act of burning was a result of subsidizing health insurance? was it more related to a common behaviour of group of people instead? | Thank you to the referee for this critical suggestion.  In responding to this inquiry, first, we double-check the pre-treatment characteristic similarity between the treated and the control group. It turns out that some of the observable characteristics are not balanced (see Panel B of Table 3). Therefore, we decide to re-estimate all of the relevant equations with the inclusion of these variables as the control variable to improve the precision. As a result, the statistical significance sifted from burning rubbish to disposing of waste in the trash can as evidence of ex-ante moral hazard. Accordingly, we revised the relevant sections in the article as the following:  (1). We added the following sentence in the introduction to improve the clarity of the connection between the approaches and the claim in conclusion (page 4; lines 74–76):  “These approaches are used to ensure that our impact estimate is a result of the subsidized insurance and not due to the common behavior of a specific group.”  (2). We add the following sentence in Section 4.2 Empirical Strategy to accentuate the importance of performing statistical matching before the estimates (page 13; lines 308–311):  “The matching strategy aims to create a comparable control group for those who own subsidized insurance. Therefore, our impact estimate will better reflect the implication of the subsidized insurance on waste management behavior and is not because of the different characteristics between the two groups.”  (3). We revised Section 5.2 in the manuscript to “5.2. Pre Estimation” and did the following revisions:  Adding Panel B in Table 3 presenting the pre- and post-matching covariates balance test.  b. Adding the following interpretation of the added part of the table (page 18; lines 414–417):  “Only household head gender is balanced between the treatment and control group but not for the rest. To anticipate bias associated with the imperfection in the matching step, we add all of the unbalanced covariates in the main estimates.”  (3). The main estimate as shown in Table 4, Table 5 and Table 6 has also been revised and we amend all the relevant interpretation of the Tables as the followings:  Page 19; lines 436–440:  “We found evidence of ex-ante moral hazard concerning the behavior of disposed waste in the trash can, but we see no impact for the other two types of outcomes. As shown in column (1) of Table 4, it is implying that 4 out of 100 insured households are less likely to dispose of waste in the trash can after receiving the treatment compared to their counterfactual.”  Page 22; lines 458–462:  “Solid waste harms human health. Household waste, in general, has contaminated water and soil in 21% and 2.7% of Indonesian villages, respectively. It has been reported that 28% of households do not have improved drinking water. In 2016, the contaminated river, which is still used for daily needs or as infiltrated groundwater, caused 17 million cases of diarrhea in Indonesia, 39.6% of which were untreated (44). ”  Page 24; lines 485–492:  “As for the outcome variable of disposing of waste to the trash can, lower-income households’ sub-population consistently to have ex-ante moral hazards, even with higher magnitudes of effects. In addition, the negative effects are also found for female-headed households, low-educated, households living in Non-Jawa Island, and households living in urban areas and population living with the presence of waste collection system. The treatment group of these sub-populations has a smaller number of households that manage waste in the range of 5.6 to 7.1 percentage points. We found the consistent null effect for outcome variables of burning trash and throwing the trash to land or open dumping.”  Last, we also revised the statement in the conclusion as the following (Page 25; lines 520–522):  “We statistically find evidence of a projection of ex-ante moral hazard of fewer people to properly dispose of their waste into the trash can after the selected households obtained the program.” |
| Another explanation about how do you relate the health insurance to a waste management would also be useful for this section. | Thank you to referees for this important suggestion. To further explain how holders of health insurance behave, we used a common grounding of health insurance's effects on health behavior. We added a new section in the manuscript, Section 2 The Competing Effects of Subsidized Health Insurance: Ex-ante Moral Hazard vs. The Improved Awareness of Hygiene. The section mainly elaborates the potential health insurance’s positive and adverse effects on hygienic lifestyle. The two competing effects explained are the conventional adverse moral hazard versus the increased awareness as a result of the increased visits to health services due to subsidized insurance. We also add a paragraph citing relevant literature that provide mixed results about the effect of subsidized insurance on hygienic behavior (see pp. 5–6). |
| In the the conclusion, the authors offer recommendation for Indonesian government that they need to advise the insured families not to burn their waste. Is it the advise you really want to take out from your study findings? I believe the authors are able to provide some more ideas to their government about waste management that they believe it relate to the provision of health insurance. | Thank you to referees for this crucial suggestion. We have added more comprehensive suggestions for the government in the Conclusion as the following:  Page 26; lines 528–542:  “The finding of this study will help the government not only focus on the curative action but more importantly on preventive ones by imposing stricter prohibitions for subsidized insurance recipients to keep their good behavior of disposing waste to trash can. However, the government needs to find more effective approaches for behavior change initiatives. Incentives are one example of the initiatives and are considered economically attractive for lower-income households. Another example is to improve household awareness of the benefits of domestic waste management. Social campaigns and marketing through various media, such as electronic, mass, and social media, are possible ways to raise awareness. Through this social dissemination, social learning is expected to occur. Households are then expected to engage more actively in waste management. The government can also increase waste sorting and recycling training for low-income households, as this will provide them with knowledge on how to make objects from recycled materials. This creates new opportunities for income-generating activities. Furthermore, social empowerment, particularly for women, can hasten the implementation of domestic waste management, including the 3R program (reuse, reduce, and recycle), and, ultimately, realizing the circular economy (zero waste)”.  Besides enforcing laws/regulation, we also suggested that the government should provide adequate waste disposal facilities to minimize improper disposal. Taking preventive action to reduce health risks will help the government to allocate its budget for health sector more effectively. We revise the paragraph as the following:  Pp. 26-27; lines 543–551:  “Due to limited lower-income household knowledge, limited waste disposal facilities, and not all households being capable of paying for private garbage collector services, households tend to dispose of their waste improperly. Households then must constantly be informed of the danger of improper waste disposal and littering as it will bring long-term impacts to their physical and environmental health. The government should also prioritize the provision of adequate waste disposal facilities across the country. The imminent reduced improper waste disposal will lead to a healthier household living environment which, in turn, will reduce the government’s potential extra budget to mitigate health-related problems due to improper waste disposal and the pollution it may cause”. |
